# Comparative Transcriptome Analysis Identifies Putative Genes Involved in the Biosynthesis of Xanthanolides in *Xanthium strumarium* L.

**DOI:** 10.3389/fpls.2016.01317

**Published:** 2016-08-30

**Authors:** Yuanjun Li, Junbo Gou, Fangfang Chen, Changfu Li, Yansheng Zhang

**Affiliations:** ^1^CAS Key Laboratory of Plant Germplasm Enhancement and Specialty Agriculture, Wuhan Botanical Garden, Chinese Academy of SciencesWuhan, China; ^2^University of Chinese Academy of SciencesBejing, China

**Keywords:** *Xanthium strumarium*, transcriptome, sesquiterpene lactone, biosynthesis, CYP450

## Abstract

*Xanthium strumarium* L. is a traditional Chinese herb belonging to the Asteraceae family. The major bioactive components of this plant are sesquiterpene lactones (STLs), which include the xanthanolides. To date, the biogenesis of xanthanolides, especially their downstream pathway, remains largely unknown. In *X. strumarium*, xanthanolides primarily accumulate in its glandular trichomes. To identify putative gene candidates involved in the biosynthesis of xanthanolides, three *X. strumarium* transcriptomes, which were derived from the young leaves of two different cultivars and the purified glandular trichomes from one of the cultivars, were constructed in this study. In total, 157 million clean reads were generated and assembled into 91,861 unigenes, of which 59,858 unigenes were successfully annotated. All the genes coding for known enzymes in the upstream pathway to the biosynthesis of xanthanolides were present in the *X. strumarium* transcriptomes. From a comparative analysis of the *X. strumarium* transcriptomes, this study identified a number of gene candidates that are putatively involved in the downstream pathway to the synthesis of xanthanolides, such as four unigenes encoding CYP71 P450s, 50 unigenes for dehydrogenases, and 27 genes for acetyltransferases. The possible functions of these four CYP71 candidates are extensively discussed. In addition, 116 transcription factors that are highly expressed in *X. strumarium* glandular trichomes were also identified. Their possible regulatory roles in the biosynthesis of STLs are discussed. The global transcriptomic data for *X. strumarium* should provide a valuable resource for further research into the biosynthesis of xanthanolides.

## Introduction

Sesquiterpene lactones (STLs) constitute a group of secondary metabolites which accumulate primarily in species of the Asteraceae family but which also occur sporadically in the Umbelliferae, Magnoliaceae and Lauraceae families (Ghantous et al., 2010). Based on carbocyclic skeletons, STLs are mainly classified into germacranolide (1), guaianolide (2), eudesmanolide (3), xanthanolide (4), elemanolide (5) and pseudo-guaianolide (6) (Supplementary Figure 1) (Fischer et al., 1979). Many STLs exhibit important pharmaceutical properties. For example, thapsigargin from *Thapsia laciniata* has been used in clinical trials to treat advanced solid tumors (Drew et al., 2013), and arglabin from *Artemisia glabella* has been shown to selectively inhibit the growth of lung, liver and ovarian cancer cells but to exert fewer side effects on normal cells (Csuk et al., 2012). Xanthanolides are bicyclic STLs in which a five carbon-membered γ-lactone ring is fused to a seven carbon-membered carbocycle (Supplementary Figure 1). The richest source of xanthanolides is found in species of the genus *Xanthium*, which has been used as a Chinese traditional herb to treat leucoderma, scrofula, herpes, sinusitis, and cancer (Saxena and Mondal, 1994; Committee, 2005; Vasas and Hohmann, 2010). Xanthanolides are believed to be the active molecules that confer the medicinal effects of *X. strumarium*. For example, several xanthanolides, such as xanthatin (7), xanthine (8), xanthumin (9), xanthinosin (10) and 8-epi-xanthatin (11), have been isolated from *X. strumarium*, and these compounds have been found to exhibit significant antimicrobial and antitumor activities (Lavault et al., 2005; Yoon et al., 2008; Nibret et al., 2011).

**FIGURE 1:**
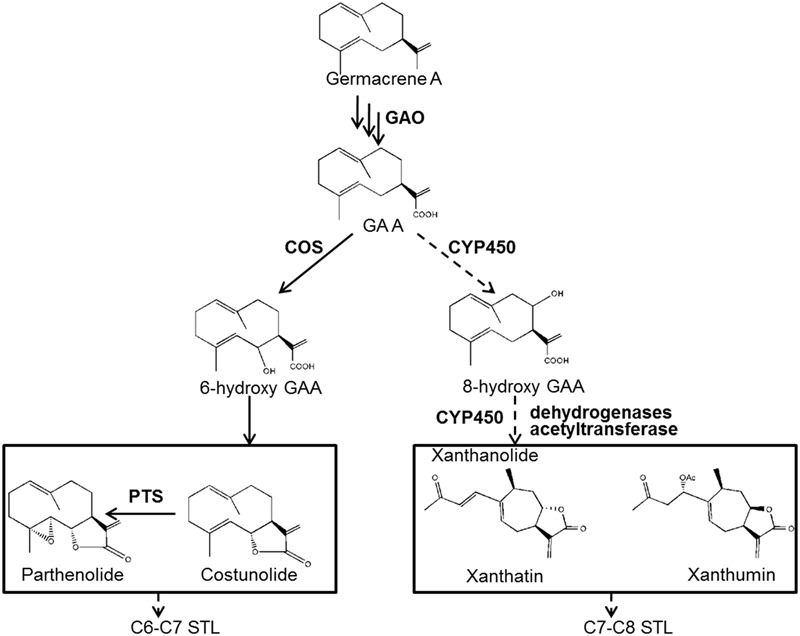
**Proposed biosynthetic pathway to the synthesis of xanthanolides.** GAO, germacrene A oxidase; COS, costunolide synthase; PTS, parthenolide synthase; GAA, germacrene A acid; STL, sesquiterpene lactone. Germacrene A is successively oxidized by the GAO at its C12 methyl group to yield GAA. GAA then can be hydrolyzed by a P450 at its C6 position for the biosynthesis of C6–C7 STLs, e.g., costunolide and parthenolide, or at its C8 position for the formation of C7–C8 end STLs, e.g., xanthanolides such as xanthatin and xanthumin. From our knowledge of the structures of xanthanolides, the biosynthetic steps from GAA to xanthanolides may involve cytochrome P450s, dehydrogenases and acetyltransferases. The pathway steps indicated by solid lines are supported by previous research, while indicated by dotted lines are proposed by this study.

Despite the multiple medicinal activities of STLs, the knowledge of STL biosynthesis remains scant. The biosynthesis of STLs derives from isopentenyl diphosphate (IPP) and dimethylallyl diphosphate (DMAPP) via the mevalonate (MVA) and/or methylerythritol 4-phosphate (MEP) pathways. IPP and DMAPP are condensed to farnesyl diphosphate (FPP) by a farnesyl diphosphate synthase (FDS), and FPP is then cyclized to various sesquiterpene skeletons by specific sesquiterpene synthases (STPs). Once sesquiterpene backbones are formed, they are then usually modified to form end STLs by tailoring enzymes. In fact, the identification of tailoring enzymes in STL biosynthesis is challenging. To expand our understanding of xanthanolide biosynthesis in *X. strumarium*, we previously performed an extensive analysis of putative STPs from *X. strumarium* by RNA sequencing, and proposed that germacrene A synthase (GAS) is the STP involved in the formation of xanthanolides (Li et al., 2016). Germacrene A undergoes a three-step sequential oxidation at its C12 methyl group to yield germacrene A acid (GAA), which is catalyzed by a cytochrome P450 enzyme, germacrene A oxidase (GAO; **Figure 1**). The GAO gene has been cloned from several plant species (Nguyen et al., 2010). GAA is a common intermediate of STL biosynthesis, and the regio-specificity of the lactone rings of STLs depends on a hydroxylation at either the C6 or C8 position of GAA, resulting in C6–C7 and C7–C8 STLs, respectively (**Figure 1**). Xanthanolides belong to the C7–C8 type of STLs, and thus their lactone rings would be facilitated by a C8-hydroxylation of GAA (**Figure 1**). The cytochrome P450 that is responsible for the formation of the C6–C7 lactone ring has been cloned from several plant species (Ikezawa et al., 2011; Liu et al., 2011, 2014), while the enzyme responsible for the formation of the C7–C8 lactone ring remains unknown. From our knowledge of the structures of xanthanolides that accumulate in *X. strumarium*, the tailoring enzymes in the biosynthesis of xanthanolides may include cytochrome P450s, dehydrogenases and acetyltransferases (**Figure 1**). To date, nothing is known about those tailoring enzymes or genes in the biosynthetic steps beyond GAA to xanthanolides.

Plant-specialized glandular trichomes are known to biosynthesize a large array of terpenoids, such as mono- and sesqui-terpenes (Lange and Turner, 2013). The glandular trichome-specificity of xanthanolide biosynthesis in *X. strumarium* has also been described recently (Chen et al., 2013). In general, a low expression of pathway genes in specialized cells is one of the major challenges to investigating plant secondary metabolism. To overcome this difficulty, the use of purified trichomes has been successfully applied to the identification of genes involved in several trichome-specific pathways (Lange and Turner, 2013). In this study – the start of a longer-term research project investigating xanthanolide biosynthesis – glandular trichomes were purified from the young leaves of one *X. strumarium* species named as Hubei-cultivar, and the glandular trichome transcriptome of this cultivar (HT) was constructed by sequencing the trichome RNA. Meanwhile, the transcriptome derived from the intact young leaves of the Hubei-cultivar (HY) was also prepared. By comparing the two transcriptomes, a number of glandular trichome-specifically expressed genes in *X. strumarium* were identified in this study. Considering that the tailoring enzymes in xanthanolide biosynthesis may include cytochrome P450s, dehydrogenases, and acetyltransferases (**Figure 1**), the putative genes encoding those enzymes that are highly expressed in *X. strumarium* glandular trichomes were extensively analyzed.

To further narrow down the number of target genes potentially involved in xanthanolide biosynthesis, another *X. strumarium* species – Zunyi-cultivar – was utilized, and the transcriptome of its young leaves (ZY) was also prepared using Illumina sequencing. The young leaves of the Zunyi-cultivar also synthesize xanthanolides (Chen et al., 2013), indicating that the gene transcripts for xanthanolide biosynthesis are present in the leaves of Zunyi-cultivar as well. Thus, in addition to the glandular trichome-specificity, the common presence of gene transcripts in both the Hubei- and Zunyi-cultivars was considered as the second filter in reducing the number of candidate genes. Eventually, a shortlist of the candidate genes, especially the cytochrome P450 genes that are potentially involved in the downstream pathway of xanthanolide biosynthesis, was identified in this study. As a starting point, this study provided a valuable basis for further elucidating the molecular mechanism underlying xanthanolide biosynthesis within the plant kingdom.

## Materials and Methods

### Plant Material

Two *X. strumarium* cultivars, designated as the Hubei- and Zunyi-cultivars, were used to construct *X. strumarium* transcriptomes. We previously reported that the young leaves of both cultivars accumulate xanthanolides (Chen et al., 2013). The *X. strumarium* seeds of the Hubei- and Zunyi-cultivars were collected from the cities of Wuhan and Zunyi of China, respectively. *X. strumarium* plants were grown in open-field conditions at the Wuhan Botanical Garden, Chinese Academy of Sciences, with appropriate irrigation and fertilization when needed.

Young leaves from 3-month-old plants were collected for the purification of glandular trichomes according to the method previously described (Chen et al., 2013). The intact young leaves and purified glandular trichomes were ground to a fine powder in liquid nitrogen, and their total RNAs were isolated for the purpose of constructing the transcriptomes. The transcriptomes derived from the Hubei-cultivar young leaves, the Zunyi-cultivar young leaves, and the Hubei-cultivar glandular trichomes were constructed, and designated as HY, ZY and HT, respectively.

### RNA Isolation and Transcriptome Construction

Total RNA was prepared using Trizol reagent and treated with RNase-free DNase I to eliminate any genomic DNA contamination. RNA quality and quantity were determined by gel electrophoresis and spectrophotometric analysis (NanoDrop ND-1000 spectrophotometer, Nanodrop Technologies, Rockland, ME, USA). Messenger RNAs were purified using magnetic oligo(dT)-attached beads and cleaved into segments; these served as templates for first-strand cDNA synthesis using random hexamer primers. The second-strand cDNA was subsequently synthesized using DNA polymerase I. Suitable lengths of cDNA fragments were purified by agarose gel electrophoresis and amplified by PCRs to make the cDNA libraries. The quality of the libraries was determined by an Agilent 2100 Bio-analyzer prior to sequencing on an Illumina Hiseq 2000 platform. For processing the sequencing data, the raw reads with adaptors and unknown nucleotides above 5%, or reads with more than 10% of bases with *Q*-value ≤ 20, were removed to generate clean reads. The clean reads were then *de novo* assembled using a Trinity software program^1^ with default parameters (Grabherr et al., 2011). The transcriptomic data have been submitted to NCBI under the accession number SRP056511.

### Functional Annotation of Unigenes

Functional annotation was performed by searching all the assembled unigenes against several public databases, using BLASTn or BLASTx with an *E*-value of 10^-5^. The public databases included the NCBI non-redundant protein (Nr), the NCBI non-redundant nucleotide (Nt), the Swiss-Prot, the Kyoto Encyclopedia of Genes and Genomes (KEGG) and the Clusters of Orthologous Groups (COG) databases. Blast2GO was used to perform gene ontology (GO) annotation of unigenes, and GO classification was then performed using WEGO software to illustrate the distribution of gene functions (Conesa et al., 2005; Ye et al., 2006).

### Identification of the Differentially Expressed Genes (DEGs) between the Trichomes and Leaves

Transcript abundance of all the unigenes was estimated by calculating the read density as “fragments per kilo-base of exon model per million mapped reads” (FPKM; Mortazavi et al., 2008). To identify the differentially expressed genes (DEGs) between the glandular trichomes and young leaves of the Hubei-cultivar, a greater than two-fold change and a false discovery rate (FDR) ≤0.001 were used to determine significant changes in expression. The DEGs were then mapped to the GO and COG databases to classify the distribution of their functions.

### Real-Time PCR Analysis

Total RNA was isolated using Trizol reagent according to the manufacturer’s instructions. First strand cDNA was synthesized using RevertAid Reverse Transcriptase (Thermo Scientific, Wilmington, DE, USA) with 1.5 μg of the total RNA as the template. The gene-specific primers used to amplify the gene transcripts are shown in Supplementary Table 1. The *X. strumarium actin 2* gene (GenBank accession no. JF434698) was used as an internal standard to normalize the variation in cDNA preparations. The qRT-PCRs were performed with an ABI 7500 Real-Time PCR Detection System using a FastStart Universal SYBR Green Mix (Roche) in three independent biological replicates with three technical repeats. The thermal cycling parameters were set as follows: one cycle at 95°C for 15 min, followed by 40 cycles at 95°C for 15 s and then 60°C for 1 min. For a given gene, the relative gene expression level was normalized with respect to the internal control *X. strumarium actin 2* gene, and calculated using the 2^-ΔΔCt^ method (Livak and Schmittgen, 2001).

## Results

### The Construction of *X. strumarium* Transcriptomes and the Evaluation of the Sequencing Depth

To globally identify potential candidate genes involved in xanthanolide biosynthesis in *X. strumarium*, transcriptomes were constructed from the young leaves of the Hubei-cultivar (HY), the young leaves of the Zunyi-cultivar (ZY), and the glandular trichomes of the Hubei-cultivar (HT), resulting in clean reads of 53,389,348 for HY, 52,073,570 for ZY, and 52,073,570 for HT (**Table 1**). All the clean reads were *de novo* assembled, using Trinity software, into 91,861 unigenes with an average length of 793 bp and N50 length of 1,238 bp. The overall GC percentage of the unigenes was about 44%, and approximately 26% of the unigenes were present in a length of more than one kilo-base (Supplementary Figure 2).

**Table 1:** Statistics of sequencing and *de novo* assembly of *X. strumarium* transcriptome.

Item	Sample	Number	Nucleotides (nt)	Valid ratio	GC (%)	N50 (bp)	Average length (bp)
Raw read	HT	56,775,466	ND	ND	ND	ND	ND
	HY	57,739,378	ND	ND	ND	ND	ND
	ZY	55,794,206	ND	ND	ND	ND	ND
Clean read	HT	52,073,570	4,686,621,300	91.7184	45.05	ND	ND
	HY	53,389,348	4,805,041,320	92.4661	44.11	ND	ND
	ZY	52,073,570	4,686,621,300	93.3315	44.00	ND	ND
Total	Unigenes	91,861	72,829,613	ND	44.39	1238	793

*Valid ratio, the numbers of clean reads/ the numbers of raw reads.*

*N50, The largest length *L* such that 50% of all nucleotides are contained in contigs (or scaffolds) of size at least *L* (de Jong et al., 2001).*

*ND, no data.*

The sequencing depth was assessed by examining the putative genes encoding enzymes in the MVA pathway, which is conserved in the plant kingdom and also is an upstream pathway to the biosynthesis of xanthanolides. The genes encoding all the MVA pathway enzymes could be identified with a full-length sequence in the database, with each transcript of the pathway being represented at a sequencing depth from 81.0 (in the case of phospho-mevalonate kinase) to 1741.3 (in the case of acetoacetyl-CoA thiolase; **Table 2**). These data suggested that the sequencing depth of the *X. strumarium* transcriptomes of this study was sufficient to enable further gene discovery.

**Table 2:** The evaluation of the sequencing depth of the *X. strumarium* transcriptome by monitoring unigenes encoding the conserved mevalonate (MVA) pathway enzymes.

The names of the MVA pathway enzymes	ID numbers of the query sequences	The highest hit in the *X. strumarium* transcriptome	Sequencing depth	Length (amino acid)
AACT	AAM00280.1	CL7008.Contig2_All (79%)	1741.2526	409
HMGS	AAD00297.1	CL4694.Contig1_All (76%)	649.8398	458
HMGR	AAA67317.1	Unigene4899_All (67%)	1305.6618	613
MVK	AED93690.1	Unigene5281_All (61%)	806.5116	387
PMK	AES72471.1	Unigene21560_All (69%)	81.0194	489
MVD	CAA76803.1	Unigene21721_All (75%)	784.0556	422

*Genes encoding all the putative MVA pathway enzymes in *X. strumarium* were identified by a tBLASTn search against the *X. strumarium* transcriptome using their respective known proteins as the query sequences.*

*AACT, acetoacetyl-CoA thiolase; HMGS, 3-hydroxy-3-methylglutaryl-CoA synthase; HMGR, 3-hydroxy-3-methylglutaryl-CoA reductase; MVK, mevalonate kinase; PMK, phospho mevalonate kinase; MVD, mevalonate diphosphate decarboxylase.*

### Functional Annotation and Classification of the *X. strumarium* Unigenes

Several publicly available databases, including Nr, Nt, Swiss-Prot, KEGG and COG, were used to annotate the unigenes (Supplementary Table 2). A total of 59,858 unigenes (65%) were annotated in the public databases. There were 57,320 unigenes (62%) matched to the Nr database with 28,954 unigenes (51%) showing significant similarities to the reference sequences from the database, with an *E*-value of less than 10^-45^. There were 34,418 unigenes (60%) showing >60% similarity with known proteins in the databases. With regard to species distribution, 82% of the unigenes could be aligned to the sequences from the top 10-hit species (Supplementary Figure 3). Surprisingly, no plant species within these top 10 hits belonged to the Asteraceae family, probably owing to the limited number of public sequences from the Asteraceae family. However, homologs of several 100 unigenes could be found in several Asteraceae species; for example, homologs of 754 unigenes (1.3%) were present in *Helianthus annuus*, and 0.4% of the unigenes could be matched to sequences from either *A. annua* or *Lactuca sativa*.

Clusters of Orthologous Groups annotation indicated that 20,275 unigenes (22%) were assigned into 25 COG categories. The most abundant category was “general function prediction only,” including 6,351 unigenes (31%), followed by the categories of “transcription” (3,802 unigenes; 19%), “replication recombination and repair” (3,500 unigenes; 17%) and “secondary metabolites biosynthesis, transport and catabolism” (1,070 unigenes; 5%; Supplementary Figure 4). GO term analysis revealed that 57,320 unigenes could be classified into three GO term categories: “biological process,” “cellular components,” and “molecular function.” The highest numbers of unigenes in the “biological process” were in the subcategories of “cellular process” and “metabolic process,” comprising 27,408 and 25,845 of the unigenes, respectively (Supplementary Figure 5). KEGG pathway analysis revealed that 34,067 unigenes (37%) could be assigned to 128 pathways. The pathways involving the highest numbers of unigenes were “metabolic pathway,” “biosynthesis of secondary metabolites,” and “plant-pathogen interaction,” comprising 7,762 (23%), 3,780 (11%) and 2,114 (6%) unigenes, respectively. In particular, 376 unigenes were assigned to the general pathway of terpenoid backbone biosynthesis, and 119 unigenes to specific pathways involved in synthesizing sesqui- and tri-terpenoids (Supplementary Table 3).

### Classification of the Trichome-Highly Expressed Unigenes

**FIGURE 2:**
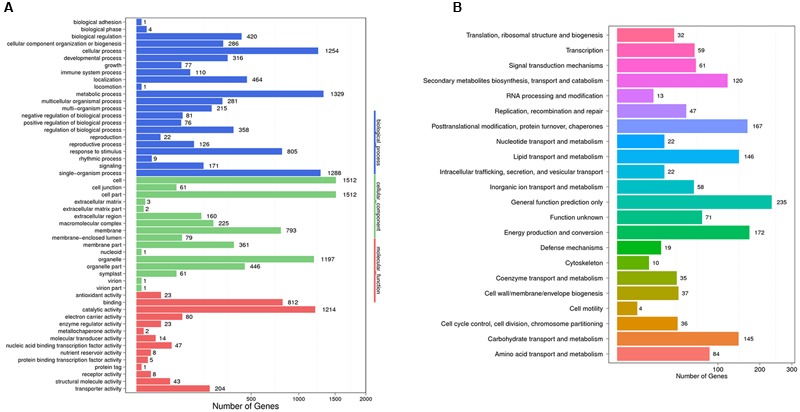
**GO annotation **(A)** and COG classification **(B)** of the 3,343 unigenes that were highly expressed in the trichomes and that were also present both Hubei- and Zunyi-cultivars.** In the “biological process” category of the GO annotation, most unigenes were assigned to the term “metabolic process” (1,329 unigenes; 40%). With a COG classification, a number of unigenes were assigned to metabolism-related processes, including 145 unigenes for the “carbohydrate transport and metabolism,” 146 for the “lipid transport and metabolism,” and 120 for the “secondary metabolites biosynthesis, transport and catabolism.”

To identify the DEGs between the trichome and leaf samples of the Hubei-cultivar, the FPKM method was used to calculate transcript abundance. A total of 3,900 unigenes showed significantly higher expression in the trichomes than in the leaves. Of these DEGs, 3,343 unigenes were expressed in both the Hubei- and Zunyi-cultivars. Based on the genetic screening criteria of this study, these 3,343 unigenes constituted the gene resource from which potential gene candidates involved in xanthanolide biosynthesis were further identified (Supplementary Table 4). From GO term analysis, the functions of these 3,343 unigenes were classified into three GO term categories: “biological process,” “cellular component” and “molecular function onthology” (**Figure 2**). In the “biological process” class, the unigenes mostly corresponded to the terms of “metabolic process” (1,329 unigenes; 40%), followed by “single-organism process” (1,288 unigenes; 39%), and “cellular process” (1,254 unigenes; 38%; **Figure 2**). COG annotation of these 3,343 unigenes showed that the category of “general function prediction only” was associated with the highest numbers (235 unigenes; 7%), followed by “energy production and conversion” (172 unigenes; 5%), “post-translation modification, protein turnover, chaperones” (167 unigenes; 5%), “carbohydrate transport and metabolism” (145 unigenes; 4%), “lipid transport and metabolism”(146 unigenes; 4%), and “secondary metabolites biosynthesis, transport and catabolism” (120 unigenes; 4%; **Figure 2**).

### The Identification of Candidate Genes in the Upstream Pathway of STL Biosynthesis

**FIGURE 3:**
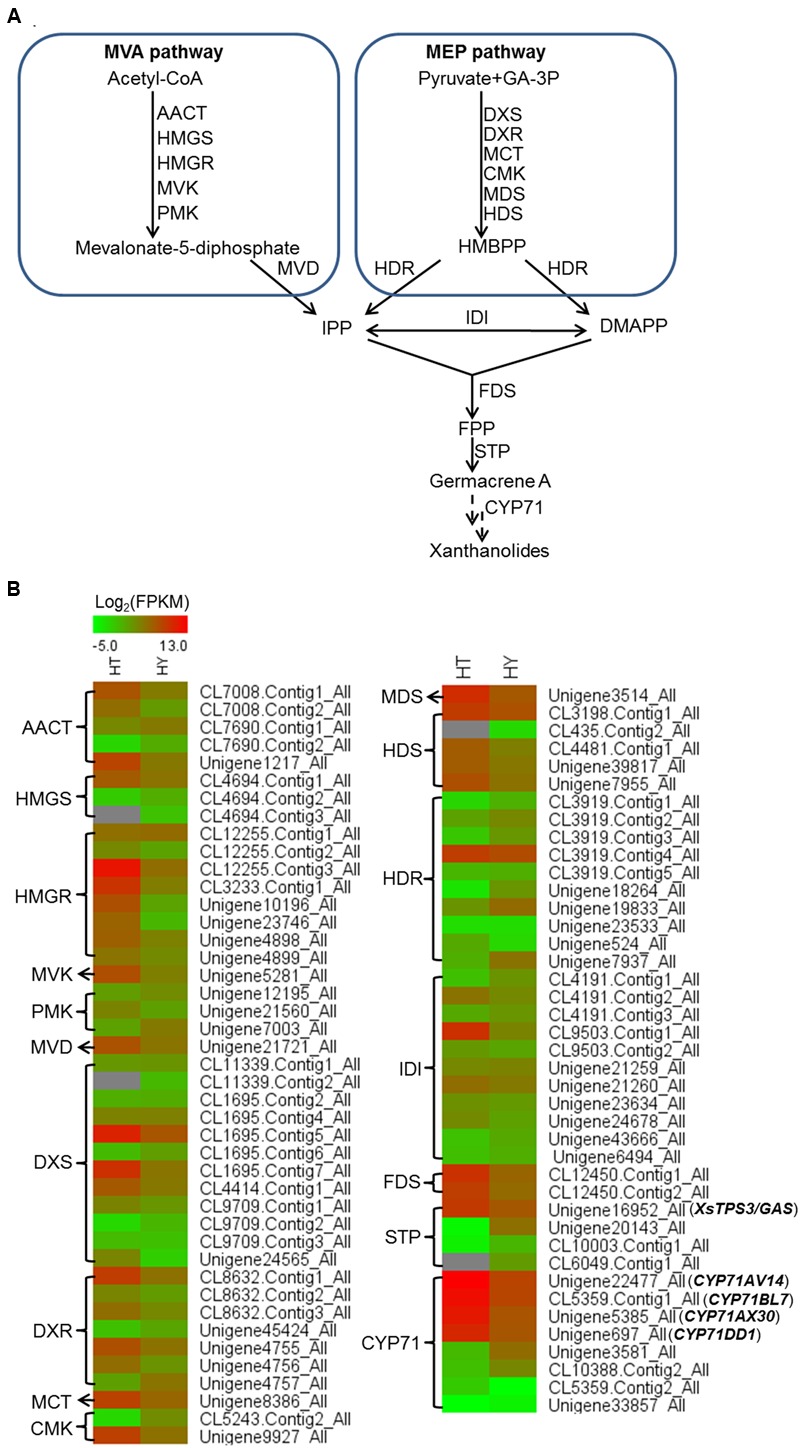
**Heatmap representing the expression of potential candidate genes involved in xanthanolide biosynthesis in the trichomes and young leaves of Hubei-cultivar. (A)** The precursors IPP and DMAPP for the biosynthesis of xanthanolides were indicated to be from both the MVA and MEP pathways by the analysis of *X. strumarium* transcriptomes. **(B)** Transcript abundances of putative pathway genes were compared between the trichomes (HT) and the young leaves (HY). The transcript abundance of genes was calculated by the FPKM method. Red color indicates high expression and green color indicates low expression. Genes encoding all the known enzymes in both the MVA and MEP pathways up to IPP or DMAPP were present in the *X. strumarium* transcriptome, and the full names of these known enzymes are shown in Supplementary Table 12. In the pathway from IPP or DMAPP to xanthanolides, there were several glandular trichome-specifically expressed unigenes, including two unigenes (CL12450.Contig1_All and CL12450.Contig2_All) encoding farnesyl diphosphate synthase (FDS), one full-length unigene (Unigene16952_All) encoding a germacrene A synthase (XsTPS3), and four full-length unigenes coding for CYP71 members (CYP71AV14, CYP71BL7, CYP71DD1, and CYP71AX30).

**FIGURE 4:**
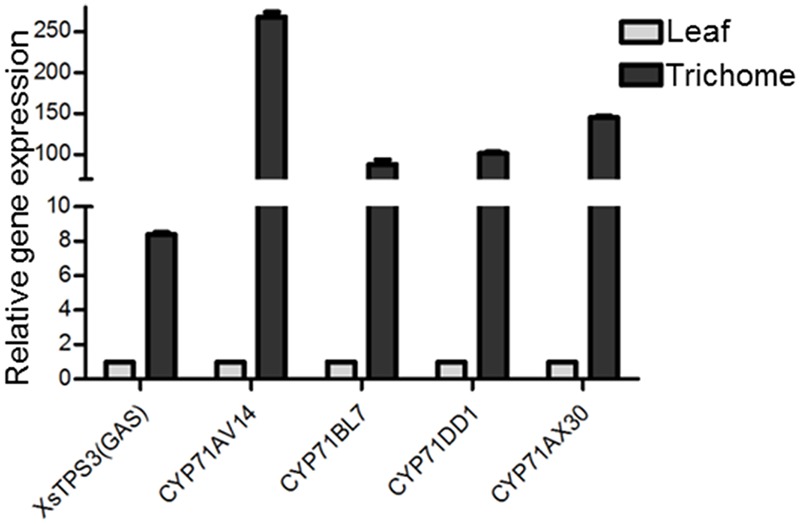
**The qRT-PCR validation of the trichome-specificity of the gene expression of the putative STP and CYP71 candidates in xanthanolide biosynthesis.** *X. strumarium actin 2* was used as an internal standard to normalize the variation in the cDNA preparations of different samples. Error bars represent mean ± SD (standard deviation) from three biological experiments with three technical replicates. The predicted amino acid sequences of these candidates are shown in Supplementary Table 9.

In the transcriptomic database, genes encoding all the known structural enzymes in the upstream pathways up to FPP were found as multiple members, including 21 members for six enzymes in the MVA pathway, 38 for seven enzymes in the MEP pathway, 11 for isopentenyl-diphosphate delta-isomerase (IDI), and two for FDS (Supplementary Table 5; **Figure 3**). Of these, 38 unigenes showed higher expression levels in the trichomes than in the leaves, with their presence in both the Hubei- and Zunyi-cultivars (**Figure 3**). Furthermore, as shown by *in silico* gene expression analysis, almost all the highest expressed member for each enzyme in the upstream pathways showed trichome-specificity (**Figure 3**), indicating that the trichome was the major site for synthesizing *X. strumarium* terpenoids. In general, FPP is cyclized to various sesquiterpene skeletons under the action of specific STPs. In the *X. strumarium* transcriptomes, four unigenes were annotated as putative full-length STPs, and they were designated as XsTPS1-4 (Supplementary Table 6). XsTPS1 had 69% amino acid identity with the germacrene D/A synthase from *Solidago canadensis*, while XsTPS2 had 70% amino acid identity to the β-caryophyllene synthase from *A. annua*. XsTPS3 was most closely related to the GAS 2 from *H. annuus* (92% amino acid identity), and XsTPS4 was related to the isocomene synthase from *Chamomile* (73% amino acid identity). Of these four STPs, only XsTPS3 (Unigene16952_All) showed trichome-specificity, meanwhile with its presence in both *X. strumarium* cultivars. The trichome-specificity for *XsTPS3* expression was further confirmed by our qRT-PCR analysis (**Figure 4**). In biochemical terms, XsTPS3 was recently proven to be a GAS, and was proposed to be the STP in xanthanolide biosynthesis in *X. strumarium* (Li et al., 2016).

### The Identification of Candidate Genes in the Downstream Pathway of STL Biosynthesis

**FIGURE 5:**
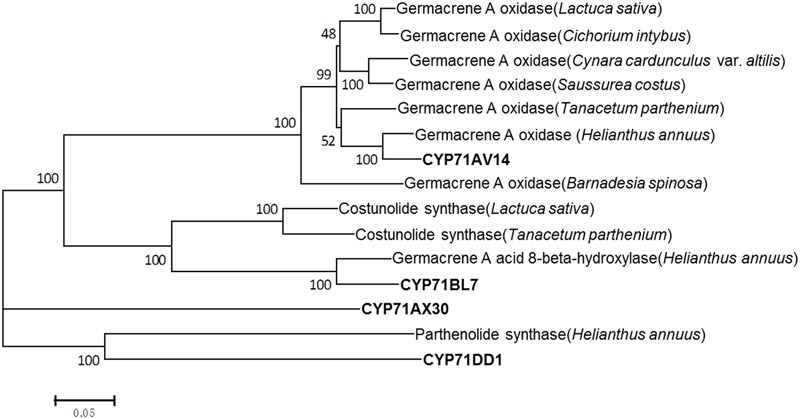
**Phylogenetic analysis of the putative CYP71 candidates of this study with the previously published known GAA or GAA-derived oxidizing enzymes.** Amino acid sequences were aligned using Clustal X software. The tree was inferred by the neighbor-joining method using MEGA 6.0 software. The scale bar represents 0.2 amino acid substitutions per site. Numbers indicate the bootstrap values of 1000 replicates. Germacrene A oxidase (*Helianthus annuus*), ADF43082.1; germacrene A oxidase (*Tanacetum parthenium*), AHN62855.1; germacrene A oxidase (*Saussurea costus*), ADF43081.1; germacrene A oxidase (*Cynara cardunculus* var. *altilis*), AIA09036.1; germacrene A oxidase (*Lactuca sativa*), ADF32078.1; germacrene A oxidase (*Cichorium intybus*), ADF43080.1; germacrene A oxidase (*Barnadesia spinosa*), ADF43083.1; costunolide synthase (*Lactuca sativa*), AEI59780.1; costunolide synthase (*Tanacetum parthenium*), AGO03790.1; germacrene A acid 8-beta-hydroxylase (*Helianthus annuus*), F8S1H3.1; parthenolide synthase (*Helianthus annuus*), AHM24033.1.

In terms of the molecular structures of xanthanolides (**Figure 1**), the downstream pathway to xanthanolides seems to involve a range of modifications including oxidation and acetylation. Oxidation could be catalyzed by cytochrome P450s or dehydrogenases while acetylation usually results from the action of acetyltransferases. In the transcriptomic data of *X. strumarium*, 397 unigenes were annotated as CYP450 (Supplementary Table 7). Among them, 70 unigenes showed higher expression in the trichomes relative to the leaves and, moreover, were present in both cultivars (Supplementary Table 7). Generally, P450s in STL biosynthesis belong to the CYP71 family. A total of 27 CYP71 candidates were found in the *X. strumarium* transcriptome (Supplementary Table 8), of which eight candidates contained complete coding sequences. Among these eight full-length CYP71 candidates, four unigenes, including candidates “Unigene22477_All,” “CL5359.Contig1_All,” “Unigene5385_All,” and “Unigene697_All,” showed trichome-specificity in their gene expression (**Figure 3**). The trichome specificity of these four CYP71 candidates was further confirmed by our qRT-PCRs (**Figure 4**). Given the fact that glandular trichomes are the primary sites for the synthesis of xanthanolides in *X. strumarium* and the currently known P450s involved in STL biosynthesis mostly belong to the CYP71 family, thus, it is likely that these four CYP71 unigenes are involved in xanthanolide biosynthesis. By the standard P450 nomenclature committee, these four CYP71 candidates have been given official names of CYP71AV14, CYP71BL7, CYP71DD1, and CYP71AX30, and their predicted amino acid sequences are shown in Supplementary Table 9. **Figure 5** shows the phylogenetic tree clustering of these four P450s with the previously published known GAA or GAA-derivative oxidizing enzymes. CYP71AV14 is closely related to the GAO from *H. annuus* (GenBank accession no. GU256646.1; **Figure 5**). GAO is a known enzyme in the pathway to STL biosynthesis (**Figure 1**), and its activity is highly conserved in the Asteraceae plant species (Nguyen et al., 2010). CYP71BL7 showed the most similarity to a GAA 8-beta-hydroxylase from *H. annuus* (HaG8H; GenBank accession no. F8S1H3.1). HaG8H catalyzes a hydroxylation of GAA at its C8 position (Ikezawa et al., 2011). Based on the proposed biosynthetic pathway of xanthanolides (**Figure 1**), a C8-hydroxylation of GAA is required for the formation of the lactone ring of C7-C8 STLs, to which xanthanolides belong. It will be of particular interest to examine whether CYP71BL7 is involved in this modification. CYP71DD1 clusters with the parthenolide synthase from *Tanacetum parthenium* (GenBank accession no. AHM24033.1, amino acid identity 45%), which catalyzes the C4–C5 double bond oxidation of costunolide to yield parthenolide (Liu et al., 2014). The oxidation of the carbon–carbon double bond was previously proposed to facilitate the STL skeleton rearrangement from germacranolides to guainolides (Piet et al., 1995). Xanthanolides contain a seven carbon guainolide carbocycle; thus CYP71DD1 may be the candidate responsible for the skeleton rearrangement in xanthanolide biosynthesis. CYP71AX30 forms a distinctive node separate from the known enzymes (**Figure 5**). By a blast search in the NR public database, the closest homolog to CYP71AX30 was revealed to be an uncharacterized *H. annuus* CYP450 (GenBank accession no. AEI59779.1).

It has been proposed that alcohol/aldehyde dehydrogenases may participate in the oxidation process in STL biosynthesis (**Figure 1**) (de Kraker et al., 2001). In the *X. strumarium* transcriptomes, 30 unigenes for alcohol dehydrogenases, and 20 for aldehyde dehydrogenases, were found in both cultivars, in addition, they showed higher expression in the trichomes than in the leaves (Supplementary Table 10). Moreover, the side chain of xanthanolides is often acetylated at the C-2 position, and this modification is usually catalyzed by acetyltransferases (**Figure 1**) (Rungeler et al., 1999). In the present study, we identified 27 acetyltransferases that showed preferential expression in the trichomes and that were also present in both cultivars (Supplementary Table 10). To date, the acetyltransferase that catalyzes the C-2 acetylation in xanthanolide biosynthesis is entirely unknown; the 27 acetyltransferases identified above are candidates for this enzyme.

### The Identification of Regulatory Genes in the *X. strumarium* Transcriptomes

An increasing number of studies are reporting an involvement of transcription factors (TFs) in regulating sesquiterpene biosynthesis (Spyropoulou et al., 2014). A total of 2,682 TFs have been identified in the *X. strumarium* transcriptomes. Analysis of gene transcript abundance has revealed that 116 TFs are highly expressed in the trichomes. Of these 116 TFs, 43 unigenes belonged to the AP2/ERF family and 20 belonged to the WRKY family. The TFs from both the AP2/ERF and WRKY families have been reported to regulate sesquiterpene biosynthesis (Xu et al., 2004; Mizoi et al., 2012). Other regulatory genes, including eight unigenes from the MYB family and seven from the NAC family, were also identified in this study (Supplementary Table 11).

## Discussion

Comparative transcriptome analysis has been successfully applied to the identification of genes involved in several secondary metabolite activities (Luo et al., 2010; Bleeker et al., 2011; Su et al., 2016). In this study, this approach was applied to identify putative genes involved in xanthanolide biosynthesis, by an extensive analysis of *X. strumarium* transcriptomes, which were derived from the young leaves of two different cultivars (HY and ZY) and the purified glandular trichomes of one of the cultivars (HT). Considering the fact that xanthanolides accumulate in both Hubei- and Zunyi-cultivars and glandular trichomes are the primary sites of xanthanolide biosynthesis (Chen et al., 2013), the rationale for screening genes involved in xanthanolide biosynthesis was as follows: first, candidate genes would be trichome-dependently expressed, and second, candidate genes would be expressed in both cultivars. Thus, comparative transcriptome analysis was performed between HY and HT, and HY and ZY.

STLs mainly accumulate in numbers of the Asteraceae family, and exhibit various pharmaceutical activities (Picman, 1986). Several STLs, such as xanthatin, xanthine, and 8-epi-xanthatin from *X. strumarium*, have been shown to exhibit significant antimicrobial, antifungal and antitumor activities (Lavault et al., 2005; Nibret et al., 2011). Generally, the biosynthesis of STLs can be divided into three stages. The first stage is the formation of the common precursor FPP. IPP and DMAPP function as the precursors for FPP biosynthesis. In the *X. strumarium* transcriptomes, both the complete plastidic MEP and the cytosolic MVA pathways for the syntheses of IPP and DMAPP were represented, with 21 unigenes corresponding to six enzymes in the MVA pathway and 38 corresponding to seven enzymes in the MEP pathway. This data suggested that the formation of FPP in the first stage in *X. strumarium* might be via both the MVA and MEP pathways. Sesquiterpene skeletons are formed in the second stage of STL biosynthesis, when FPP is converted to specialized sesquiterpene backbones by specific STPs. Germacrene A synthase was proposed to be the STP in the pathways to the synthesis of STLs such as guaianolide, eudesmanolide and xanthanolide (Parodi and Fischer, 1986; Piet et al., 1995; de Kraker et al., 1998). Eleven unigenes were annotated as STPs in the *X. strumarium* transcriptomes, and only one of these STPs (Unigene16952_All) met the gene screening criteria of this study, which was that the genes of interest should be trichome-dependently expressed and should be presented in both cultivars. Database searches revealed that Unigene16952_All (XsTPS3) is most homologous to the GAS from *H. annuus*, and its germacrene A-forming activity was recently confirmed by heterologous expression (Li et al., 2016). Thus, the STP candidate outcome from the analysis of the *X. strumarium* transcriptome suggested that the gene screening criteria of this study were useful for reducing the number of candidate genes in xanthanolide biosynthesis. In the third stage, germacrene A is then modified to form xanthanolides by various tailoring enzymes such as cytochrome P450s, dehydrogenases, and acetyltransferases (**Figure 1**).

Cytochrome P450s are the most important type of modifying catalyst and perform many oxidations in STL biosynthetic pathways. Based on the gene expression analysis of the *X. strumarium* transcriptomes, we found that 70 putative cytochrome P450s were highly expressed in the *X. strumarium* trichomes. To date, the P450 enzymes identified in STL biosynthesis are primarily CYP71 P450s, and thus we focused much of our attention on members of this family. An intriguing result was that the top four most highly expressed cytochrome P450s (CYP71AV14, CYP71BL7, CYP71DD1, and CYP71AX30) in the trichome library were all found to belong to the CYP71 family. By a blast search, CYP71AV14 was found to show most homology with *H. annuus* germacrene A oxidase (HaGAO; Nguyen et al., 2010). GAO catalyzes three-step oxidations at the C12 position of germacrene A to form GAA (Nguyen et al., 2010). GAA is a common intermediate in the formation of the lactone moiety of STLs, and can be hydrolyzed by a P450 at its C6 position to form a C6–C7 lactone, e.g., in the formation of costunolide (de Kraker et al., 2002; Ikezawa et al., 2011), or at its C8 position to form a C7–C8 lactone (**Figure 1**). Recently, the P450 that synthesizes the C6–C7 lactone from GAA has been characterized from several plant species (Ikezawa et al., 2011; Liu et al., 2014). The *X. strumarium* STLs belong to the class of C7–C8 lactones, and thus their biosynthesis requires a C8-hydroxylation of GAA. The C7–C8 lactone-forming P450s have not yet been isolated from any plant species. A sunflower P450 (HaG8H) was recently reported to be capable of hydrolyzing GAA at its C8 position, but that study found that the enzyme could not further convert the hydrolyzed GAA into a C7–C8 lactone (Ikezawa et al., 2011). Interestingly, a BLAST analysis of CYP71BL7 revealed that the closest match found from public database searches was with the sunflower HaG8H, an enzyme capable of hydrolyzing the GAA at its C8 position. This information may suggest that CYP71BL7 is the most likely candidate responsible for the formation of the C7–C8 lactone group. The P450 candidate CYP71DD1 showed highest similarity with an uncharacterized P450 from *H. annuus* (GenBank accession no. AEI59778.1; amino acid identity 78%), followed by the parthenolide synthase from *T. parthenium* (GenBank accession no. AHM24033.1; identity 45%). Parthenolide synthase catalyzes the C4–C5 double bond oxidation of costunolide to form parthenolide, and the gene coding for this enzyme has recently been isolated from *T. parthenium* (Liu et al., 2014). Apparently, from our knowledge of the structures of *X. strumarium* xanthanolides (Supplementary Figure 1), we construed that the double bond epoxidation may also apply to the biosynthesis of xanthanolides, this tempted us to speculate that the P450 candidate CYP71DD1 would be the candidate for this modifying process. The P450 CYP71AX30 showed the most similarity with a *H. annuus* cytochrome P450 of unknown function (GenBank accession no. AEI59779.1, 80% identity), and it was not possible to deduce its function at this stage. In addition to cytochrome P450s, we also analyzed other modifying enzymes such as aldehyde/alcohol dehydrogenases and acetyltransferases; and their respective possible candidates for xanthanolide biosynthesis are shown in Supplementary Table 10. These enzymes showed the trichome-specificity and, additionally, were expressed in both *X. strumarium* cultivars.

The mechanism underlying the regulation of STL biosynthesis is largely unknown. A total of 2,679 unigenes (3%) encoded TFs, of which 116 TFs met the gene screening criteria of this study. Of these 116 TFs, 43 TFs were classified into the AP2/ERF family and 20 into the WRKY family. Members of TFs belonging to the AP2, WRKY, or MYC family were previously reported to play regulatory roles in terpenoid biosynthesis (Spyropoulou et al., 2014). For example, jasmonic acid (JA)-responsive AP2 family TFs are purported to regulate artemisinin biosynthesis in *A. annua* (Yu et al., 2012), and one WRKY member is purported to participate in regulating cotton sesquiterpene biosynthesis (Xu et al., 2004). Thus, an area that warrants further investigation is whether the TFs described above play regulatory roles in xanthanolide biosynthesis.

## Conclusion

A comprehensive transcriptomic database of *X. strumarium* was constructed from the young leaves of the two *X. strumarium* cultivars, and from the purified glandular trichomes of one of the cultivars using an Illumina HiSeq2000 sequencer. From the comparative transcriptome analysis, a number of putative genes involved in xanthanolide biosynthesis – especially those encoding tailoring enzymes in their downstream pathway – were identified. Our study provides an effective basis from which to reveal the enzymes that catalyze the oxidizing and acyl-transferring steps in xanthanolide biogenesis in *X. strumarium*.

## Author Contributions

YZ designed this project; YL and FC performed the experiments and analyzed the data; JG and CL provided assistance in collecting plant materials; YL and YZ wrote the manuscript.

## Conflict of Interest Statement

The authors declare that the research was conducted in the absence of any commercial or financial relationships that could be construed as a potential conflict of interest.

## Footnotes

1 http://trinityrnaseq.sourceforge.net/
