# Supplementary material for: Comparative Transcriptome Analysis Identifies Putative Genes Involved in the Biosynthesis of Xanthanolides in Xanthium strumarium L
Source: Front Plant Sci. 2016 Aug 30;7:1317. doi: 10.3389/fpls.2016.01317 (PMC5003840; doi:10.3389/fpls.2016.01317)
Supplement: Supplementary file 1 [file Data_Sheet_1.ZIP › Supplemental data/Supplementary Figures.docx]

Supplementary Material

**Comparative transcriptome analysis identifies putative genes involved in xanthanolide biosynthesis in *Xanthium strumarium* L.**

**Yuanjun Li^1，2^, Junbo Gou^1，2^, Fangfang Chen^1^, Changfu Li^1^, Yansheng Zhang^1*^**

^1^CAS Key Laboratory of Plant Germplasm Enhancement and Specialty Agriculture, Wuhan Botanical Garden, Chinese Academy of Sciences, Wuhan, Hubei, China

^2^ University of Chinese Academy of Sciences，Bejing 100049, China

*** Correspondence:** Yansheng Zhang: [zhangys@wbgcas.cn](mailto:zhangys@wbgcas.cn)

## Supplementary Figures: 6

**
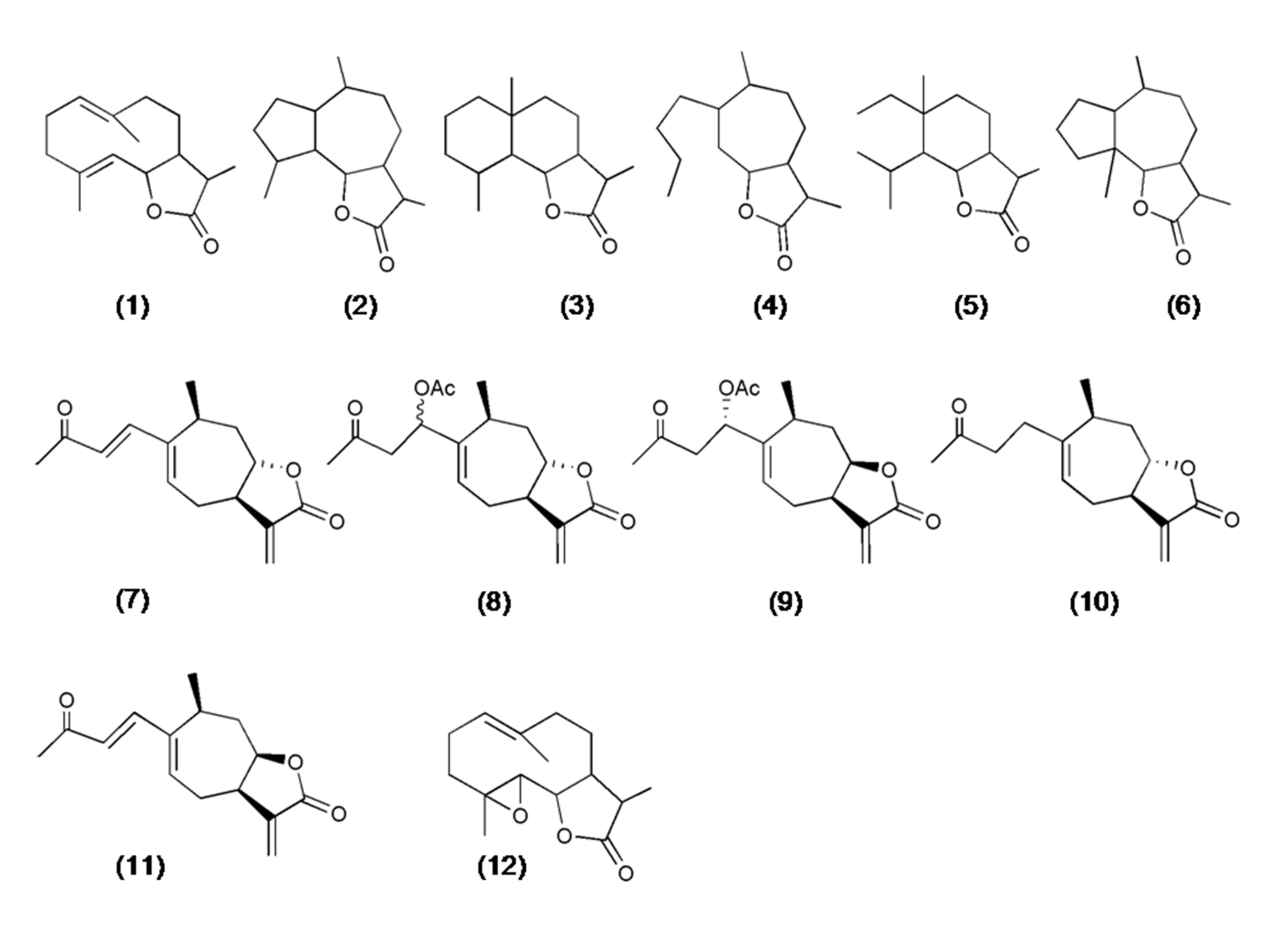
**

**Supplementary Figure 1.The structures of representative sesquiterpene lactones mentioned in the main manuscript**. The compounds were numbered according to those mentioned in main manuscript.

**
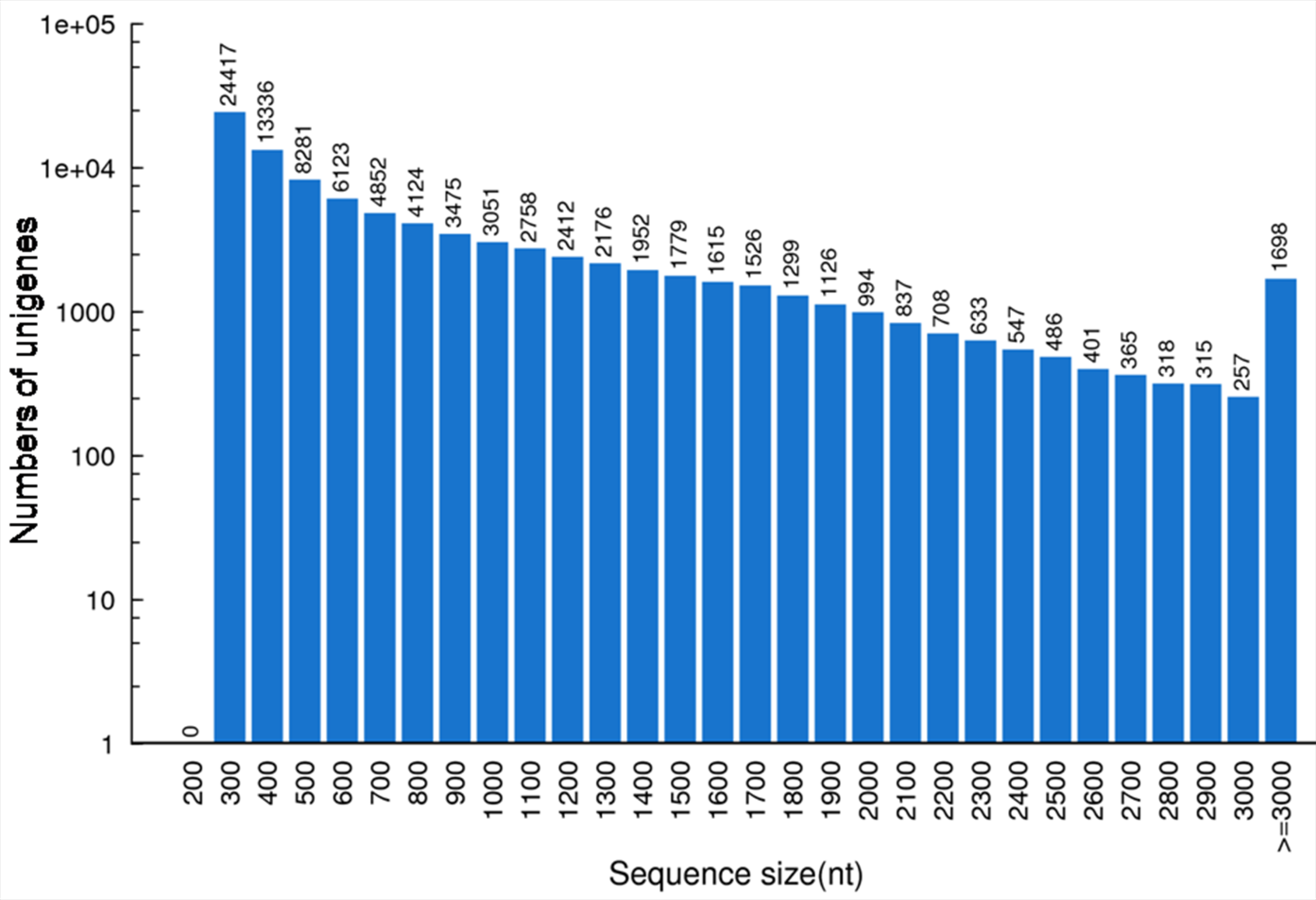
**

**Supplementary Figure 2. Length distribution of all the *X. strumarium* unigenes in the transcriptome database.**

**
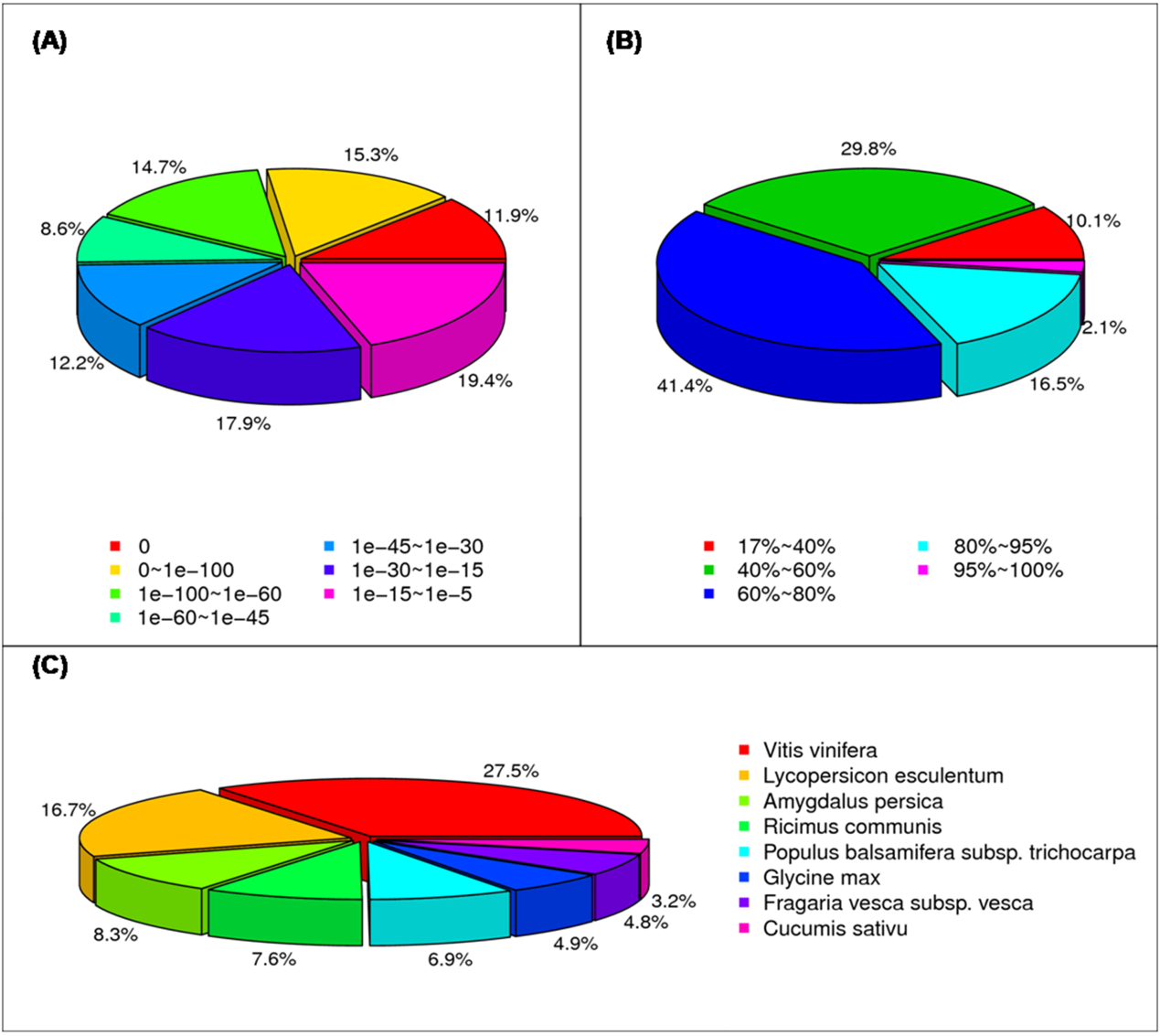
**

**Supplementary Figure 3.** (**A**) The E-value distribution of the result of NR annotation; (**B**) The similarity distribution of the result of NR annotation; (**C**) The species distribution of the result of NR annotation.

**
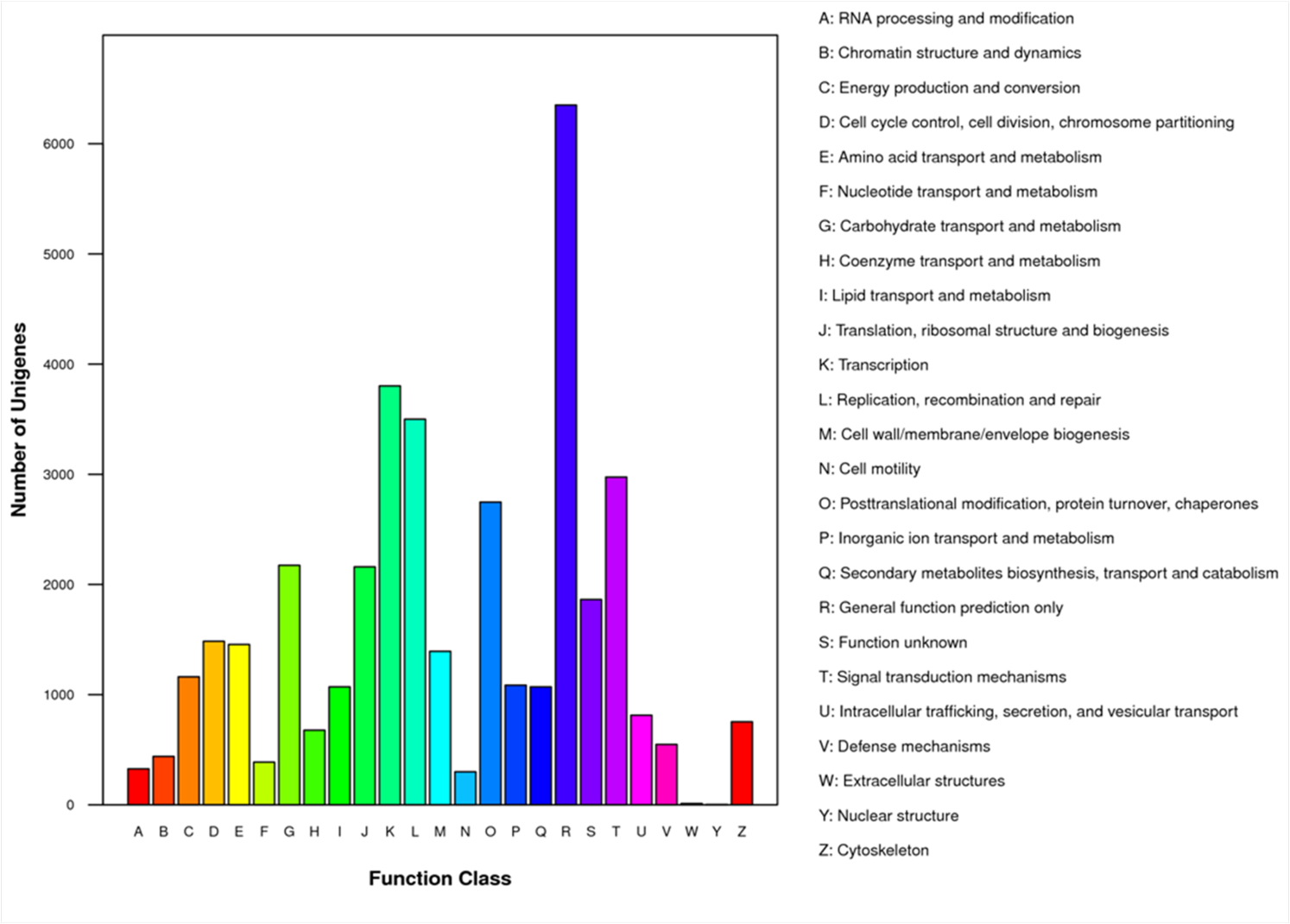
**

**Supplementary Figure 4. COG classification of all the *X. strumarium* unigenes*.*** 20,275 contigs were classified into 25 COG categories. The X-axis indicates a variety of functional COG terms, which are showed by letter A-Z and annotated besides the figure. The Y-axis shows the number of unigenes in each COG term.

**
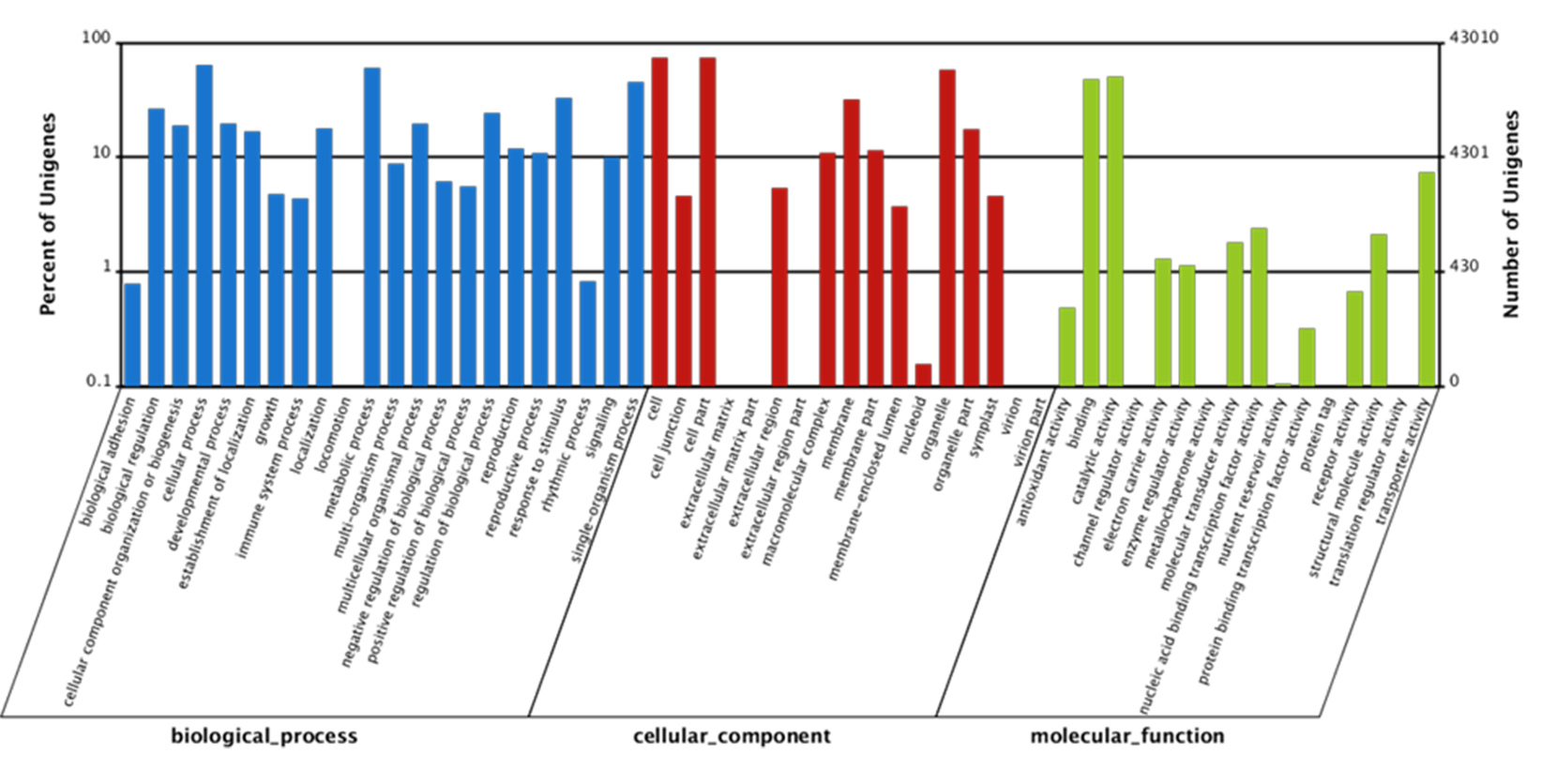
**

**Supplementary Figure 5. Distribution of GO annotation of all the *X. strumarium* unigenes**. The GO annotation were classified into three main categories: biological process, cellular component, and molecular function. The left Y-axis indicates the percentage of a specific category of genes in that category. The right Y-axis shows the number of genes in a category.
