# Supplementary material for: Comparative Transcriptome Analysis Identifies Putative Genes Involved in the Biosynthesis of Xanthanolides in Xanthium strumarium L
Source: Front Plant Sci. 2016 Aug 30;7:1317. doi: 10.3389/fpls.2016.01317 (PMC5003840; doi:10.3389/fpls.2016.01317)
Supplement: Supplementary file 1 [file Data_Sheet_1.ZIP › Supplemental data/Supplementary Table 1.docx]

**Supplementary Table 1. Primers used for the qRT-PCRs of this study.**

| Number of primers | Names of the primers | Sequences of the primers | Descriptions |
| --- | --- | --- | --- |
| 1 | XsTPS3-RT-FP | TCCGCCTTCTAACCCATGCGATAA | for amplifying XsTPS3 transcripts in qRT-PCR |
| 2 | XsTPS3-RT-RP | GCAATGTCTTGGAACGCTTCTTTT | for amplifying XsTPS3 transcripts in qRT-PCR |
| 3 | XsCYP1-RT-FP | ACCTGCCCTACTTAAACCTTGTGGT | for amplifying XsCYP1 transcripts in qRT-PCR |
| 4 | XsCYP1-RT-RP | TCTGCGACTTTCAAAATAGGGGCTT | for amplifying XsCYP1 transcripts in qRT-PCR |
| 5 | XsCYP2-RT-FP | TTGGAGGAACATAGAAGTAGAACAC | for amplifying XsCYP2 transcripts in qRT-PCR |
| 6 | XsCYP2-RT-RP | GAAGACGAACTTGTCCCAGC | for amplifying XsCYP2 transcripts in qRT-PCR |
| 7 | XsCYP3-RT-FP | CTATCCAGAGCGGTTTGAAAAT | for amplifying XsCYP3 transcripts in qRT-PCR |
| 8 | XsCYP3-RT-RP | TTGGCAACAAGTAAGCGACC | for amplifying XsCYP3 transcripts in qRT-PCR |
| 9 | XsCYP4-RT-FP | CCCGTGAATCAACCAAAGAA | for amplifying XsCYP4 transcripts in qRT-PCR |
| 10 | XsCYP4-RT-RP | TGCGAAACTAATGCCAGGAC | for amplifying XsCYP4 transcripts in qRT-PCR |
| 11 | Actin 2-RT-FP | TACTACAACGGCAGAACGGGAAA | for amplifying *X. strumarium* actin 2 transcripts in qRT-PCR |
| 12 | Actin 2-RT-RP | TCATAGACGGCTGGAACAAAACC | for amplifying *X. strumarium* actin 2 transcripts in qRT-PCR |
