# Supplementary material for: Comparative Transcriptome Analysis Identifies Putative Genes Involved in the Biosynthesis of Xanthanolides in Xanthium strumarium L
Source: Front Plant Sci. 2016 Aug 30;7:1317. doi: 10.3389/fpls.2016.01317 (PMC5003840; doi:10.3389/fpls.2016.01317)
Supplement: Supplementary file 1 [file Data_Sheet_1.ZIP › Supplemental data/Supplementary Table 12.docx]

**Supplementary Table 12. The full names for the abbreviations used in the figure 3 of the main text.**

AACT, acetoacetyl-CoA thiolase;

CMK, 4-(cytidine 5’-diphospho)-2-*C*-methyl-D-erythritol kinase;

DMAPP, dimethylallyl diphosphate;

DXR, 1-deoxy-D-xylulose-5-phosphate reductoisomerase;

DXS, 1-deoxy-D-xylulose-5-phosphate synthase;

FDS , farnesyl diphosphate synthase;

GA-3P, glyceraldehyde-3-phosphate;

HDR, (*E*)-4-hydroxy-3-methylbut-2-enyl diphosphatereductase;

HDS, (*E*)-4-hydroxy-3-methylbut-2-enyl diphosphate synthase;

HMGR, 3-hydroxy-3-methylglutaryl-CoA reductase;

HMGS, 3-hydroxy-3-methylglutaryl-CoA synthase;

IDI, isopentenyl-diphosphate delta-isomerase;

IPP , isopentenyl diphosphate;

MCT, 2-*C*-methyl-D-erythritol-4-phosphate cytidylyltransferase;

MDS, 2- *C*-methyl-D-erythritol-2,4-cyclodiphosphate synthase;

MVD, mevalonate diphosphate decarboxylase;

MVK, mevalonate kinase;

PMK, phospho mevalonate kinase;

STP, sesquiterpene synthase;
