# Supplementary material for: Comparative Transcriptome Analysis Identifies Putative Genes Involved in the Biosynthesis of Xanthanolides in Xanthium strumarium L
Source: Front Plant Sci. 2016 Aug 30;7:1317. doi: 10.3389/fpls.2016.01317 (PMC5003840; doi:10.3389/fpls.2016.01317)
Supplement: Supplementary file 1 [file Data_Sheet_1.ZIP › Supplemental data/Supplementary Table 2.docx]

**Supplementary Table 2. Statistics of annotations for assembled unigenes in *X. strumarium* in different public databases.**

| Category | Account | Percentage^a^ |
| --- | --- | --- |
| NR | 57,320 | 62.4% |
| NT | 43,376 | 47.3% |
| Swiss-Prot | 38,311 | 41.7% |
| KEGG | 34,067 | 37.1% |
| COG | 20,275 | 22.1% |
| GO | 43,010 | 46.8% |
| All | 59,858 |  |

^a^ The percent of annotated unigenes in the total of 91,861 assembled unigenes.
