# Supplementary material for: Comparative Transcriptome Analysis Identifies Putative Genes Involved in the Biosynthesis of Xanthanolides in Xanthium strumarium L
Source: Front Plant Sci. 2016 Aug 30;7:1317. doi: 10.3389/fpls.2016.01317 (PMC5003840; doi:10.3389/fpls.2016.01317)
Supplement: Supplementary file 1 [file Data_Sheet_1.ZIP › Supplemental data/Supplementary Table 9.docx]

**Supplementary Table 9. The predicted amino acid sequences of the putative STP and CYP450 candidates in xanthanolide biosynthesis**

XsTPS3

M A A V G A N A T L L T N T K S T V E P V R P L A N F P P S V W G D M F L S F S L D N S K M E E Y A K A M E K P K Q E V R R L I L D P T M D S N K K L S L I Y V V H R L G L T Y M F L K E I E G Q L D R L F E E F N L E D Y V D V D L H T I S I N F Q A F R H L G Y K L P C D V F N K F K N N D S N A F K E S I A S D V R G L L G L Y E S A Q L R V K G E K I L D D A S A F A E T K L K S L V N T L E G S L A Q Q V K Q A L K R P F H Q G M P M V E A R L Y F T N Y Q E E F S K Y D S L L K L A K L H F N Y L Q L Q Q K E E L R I V S K W W K D M R F Q E T T P Y I R D R V P E I Y L W I L G L Y F E P K Y S L A R I I A T K I T L F L V V L D D T Y D A Y G T L E E L R L L T H A I N R W D M R A M S D I P E Y I R P F Y K I L L D E Y A E L E K Q L A K E G R L K S V I A S K E A F Q D I A R G Y I E E A E W T N S G Y V A S F P E Y M K N G L I T S A Y N V I S K S A L V G M G E V V S A D A L A W Y E S H P K I L Q A S E L I S R L Q D D V M T Y Q F E R E R G Q S A T G V D S Y I K T Y G V S E K E A I E E L K K M I E N A W K D I N E G C L K P R E V S M D L L A P I L N L A R M I D V V Y R Y D D G F T F P G K T L K E Y I T L L F V D S L P M

CYP71AV14

M E V S L T T S I A L A T I V F F L Y K L A T R P T S I K N R L P E P W R L P I I G H M H H L I G T L P H R G V M D L A R K Y G S L M H L Q L G E V S A I V V S S P K W A K E I L T T Y D I P F A N R P E T L T G E I V A Y H N T D I V L A P Y G E Y W R Q L R K L C T L E L L S V K K V K S F Q S V R E E E C W N L V Q E I K A S G S G T P F N L S E G I F K L V A T V L S R A A F G K G I K D Q K E F T E I V K E I L R Q T G G F D V A D I F P S K K F L H H L S G K R G R L T S I H N K L D S L I N N L I A E H T V N S S N K T N E T L L D V L L R L K D S E D F P L T A D N V K A I I L D M F G A G T D T S S A T V E W A I S E L I R C P R A M E K V Q A E L R Q A L K G K E K I K E E D I Q D L P Y L N L V V R E T L R L H P P L P L V M P R E C R Q P M N L A G Y D V A N K T K L I V N V F A I N R D P E Y W K D A E S F I P E R F E K S N T T I M G A D Y E Y L P F G A G R R M C P G S A L G L A N V Q L P L A N I L Y Y F K W Q L P N G A S N D Q L D M T E S F G A T V Q R K T G L I L V P S Y

CYP71BL7

M E L F T I F T I V T S S L I L F I C W A L I T T K V P K N L P P G P P K L P I I G N I H L L D K I A P H R N L R N L A R K Y G P I M H L Q L G Q V S T V V I S S P R L A H E I L K T Q D L S F A D R P T I T T S Q I F F Y K A S N I A A A R Y G N Y W R Q M K K I C T L E L L S A K K S R S F F Y I R E E E L T R T F K F L E S S S G T P I I L R D T I Q E M V N N V V S R A T L G D I S Q D R Q F I I D A T Y T M L K S F N S F N L F N Y Y P S L S F F N V I F G K K A E W L K V H K E V D V I L E K V L E E H R S R T R D K H D H E D L V D V L L R V K R T G D L D M P I T D D H I K A I I L E M L T A G T S S S S M T I E W A F T E M M R N P E V M K K A Q S E V R A V A K G D R V T E A D L Q S L D Y T K L V I K E T L R L H G V P I L V P R E N Q E D C V V N G Y D I P A K T R I L V N A W A C A T D P D S W E D P D S F I P E R F E N N P I N Y S G T D F E F I P F G A G R R I C P G M N F G M G T V E Y V V A N L L H H F D W K L P D G V K P H D I D T S E I T G I S T L P I H P L K M V P I S L S K S N

CYP71DD1

M L T F F P P W L L P T V V I L T I S Y I M L W K K P S K G A S G P N P P P G P P G L P I I G N L H Q F H G K N F H E T S Y E L A Q K Y G P I I Q V H L G S Q P I V I I S S S E L A N E A F K T H D H V L V N R A Y S D N L R Q L T F D Y N E I A W V P Y G D H W K F M R K M L V N D F L N S K L M S K S F A K A L D M E V K S M L D N L P Y G T V T N L N N V L G N F V C D F I S R V V A G K S Y R E V K I K G K P M K E I L D E M I V F F S G S F S E M F P K Y G W I L E V L S G W T R R V D K H M A N F N E L L E M M I D E H T D H T S E E E K D M I D V C R S L L S R V E M K A I M S T A L N G A I D T T L L T L L W A M S E I A K N P R I M H K L Q H E I R S C S G N K E R L D E T D T S K M T Y L K Y V V K E T L R C H A P P P F T I P R G C P S H I K I G G Y D I L P G T K V L I S T W A I G K D P K V W T E N A H E F Y P E R F E N L V L E Q F D M A P F G G G R R T C P G N K F A T I N I E V V I A N L L Y K I D W K L P P G L T T K D L N M E E E G R L L V A K K T P L Y L V P I K H N S K A K

CYP71AX30

M F L A L Q V F L F S S L P L L V A I L L L K S Y Y F S S K S H L N L P P S P P K L P L I G N F H Q L G S G S H R F L Q S M A K T Y G P L M M I H I G S V P V L V A S S V D A A R E I M K T H D V I F S D R P F I S T A D R L F Y G S K D I A F C K Y G E Y W R Q V K S I S V L H L L S N K R V K S Y Y Q V R E D E V A H L I K K I Q E A N K S V V N M S D L L L S L T N N V I C R V A L G K T Y E G K E A N Y K N S L D Q I G E M L G H I S I G S Y I P W L A W V D K L S G L H G K A D K L A K E I D D F Y E I V I D E H M N K K N F G D Q G Q D L V D I L L E V Q R E N S T G F L L E R Y M I K A I I M D I F G A G T D T I F A S L G W A F S E I L R N P R T M K K L Q Q E A R K I G Q G R S M I P E D D I V K M P Y L K A I L K E A F R L H T P A P L L V P R E S T K E V K L F G Y D I P A G T Q V M I N A W A I A R D P S I W E E P D E F R P E R F L N N R M D Y R G L D F E L I P F G A G R R I C P G I S F A E A I I E Y A L A N L V Y K F D F S L P P E G L D M T E G D G I T V H R K F P I H V I A T P S K
